# In vitro mineral nutrition of *Curcuma longa* L. affects production of volatile compounds in rhizomes after transfer to the greenhouse

**DOI:** 10.1186/s12870-018-1345-y

**Published:** 2018-06-18

**Authors:** Rabia F. El-Hawaz, Mary H. Grace, Alan Janbey, Mary Ann Lila, Jeffrey W. Adelberg

**Affiliations:** 1 Department of Plant and Environmental Sciences, Clemson University, Clemson, SC 29634 USA; 2 Plants for Human Health Institute, North Carolina State University, Kannapolis, NC 28081 USA

**Keywords:** Bioreactor, Sesquiterpenes, GC-MS, *Curcuma longa* rhizome, Response surface methods, Fed-batch technique

## Abstract

**Background:**

Turmeric is a rich source of bioactive compounds useful in both medicine and cuisine. Mineral concentrations effects (PO_4_^3−^, Ca^2+^, Mg^2+^, and KNO_3_) were tested during in vitro rhizome development on the ex vitro content of volatile constituents in rhizomes after 6 months in the greenhouse. A response surface method (D-optimal criteria) was repeated in both high and low-input fertilizer treatments. Control plants were grown on Murashige and Skoog (MS) medium, acclimatized in the greenhouse and grown in the field. The volatile constituents were investigated by GC-MS.

**Results:**

The total content of volatiles was affected by fertilizer treatments, and in vitro treatment with Ca^2+^ and KNO_3_; but PO_4_^3−^ and Mg^2+^ had no significant effect. The content was higher in the high-input fertilizer treatments (49.7 ± 9 mg/g DM) with 4 mM Ca^2+^, 60 mM KNO_3_ and 5 mM NH_4_^+^, than the low-input fertilizer (26.6 ± 9 mg/g DM), and the MS control (15.28 ± 2.7 mg/g DM; 3 mM Ca^2+^, 20 mM K^+^, 39 mM NO_3_^−^, 20 mM NH_4_^+^, 1.25 mM PO_4_^3−^, and 1.5 mM Mg^2+^). The interaction of Ca^2+^ with KNO_3_ affected curcumenol isomer I and II, germacrone, isocurcumenol, and β-elemenone content. Increasing in vitro phosphate concentration to 6.25 mM increased ex vitro neocurdione and methenolone contents.

**Conclusion:**

These results show that minerals in the in vitro bioreactor medium during rhizome development affected biosynthesis of turmeric volatile components after transfer to the greenhouse six months later. The multi-factor design identified 1) nutrient regulation of specific components within unique phytochemical profile for *Curcuma longa* L. clone 35–1 and 2) the varied phytochemical profiles were maintained with integrity during the greenhouse growth in high fertility conditions.

**Electronic supplementary material:**

The online version of this article (10.1186/s12870-018-1345-y) contains supplementary material, which is available to authorized users.

## Background

The variety of active ingredients has made turmeric, *Curcuma longa* an important medicinal herb, where the crude extract has many therapeutic properties such as anti-microbial, anti-inflammatory [1], parasiticidal activity [2], and hypoglycemic effects [3]. Volatile components derived from turmeric is a raw material of culinary, drug, and cosmetic industries. Following the phenolic components, curcuminoids; terpenoids are the second major bioactive constituents of *Curcuma* species. Terpenoids occur in a large variety of mono- and sesquiterpenes in *Curcuma* [4]. Their synthesis in leaves and the accumulation of these compounds in plant tissue are affected by biotic and abiotic factors [5–8]. One of the problems associated with turmeric cultivation is that the content of curcumin, volatile components, and oleoresin vary with environmental factors that affect the economic value of the crop [9–11].

In vitro cultures were recognized as potential methods to produce secondary metabolites from plant cells, tissues, and organs for industry [12, 13]. A large mass of plant material is a primary requirement to produce chemical compounds [14] and increasing the plant mass could be achieved by either using large vessels in “bioreactors” with liquid media [15, 16] or adding more initial plants mass with fed-batch supplementation during the culture cycle [17]. Commercial scale fed-batch bioreactors (10,000 to 20,000 L) used to produce ginseng saponins from *Panax ginseng* root culture have achieved high yield [18]. Increasing in vitro propagated biomass in a field or greenhouse poses an alternative to upscale medicinal chemistry while maintaining quality attributes obtained in the bioreactor [19].

In vitro treatments including minerals, plant density, and fed-batch techniques (in vitro treatments) applied during 5 months of micropropagation in fed-batch bioreactors [17] effects the plant quality during the subsequent 6 months of greenhouse growth where rhizomes continue to attain mass. These effects include both the relative fresh mass gain of nursery plants [20] and the concentration of curcuminoids in the rhizome following a season of growth [19]. Following the previous work, this current paper investigated the effects of the in vitro treatments and fertilizer treatments on the GC-MS profile and content of *C. longa* rhizomes in the greenhouse by using a multi-factor response surface method (RSM).

## Methods

### Plant materials

*Curcuma longa* L35–1 rhizomes were provided by University of Arizona Southwest Center for Natural Products Research and Commercialization (UA Herbarium #375,742, ARIZ) and used for the GC-MS analysis. The stock plants were initiated and propagated as described in [15].

### Micropropagation in bioreactor and fed-batch technique

**Fig. 1:**
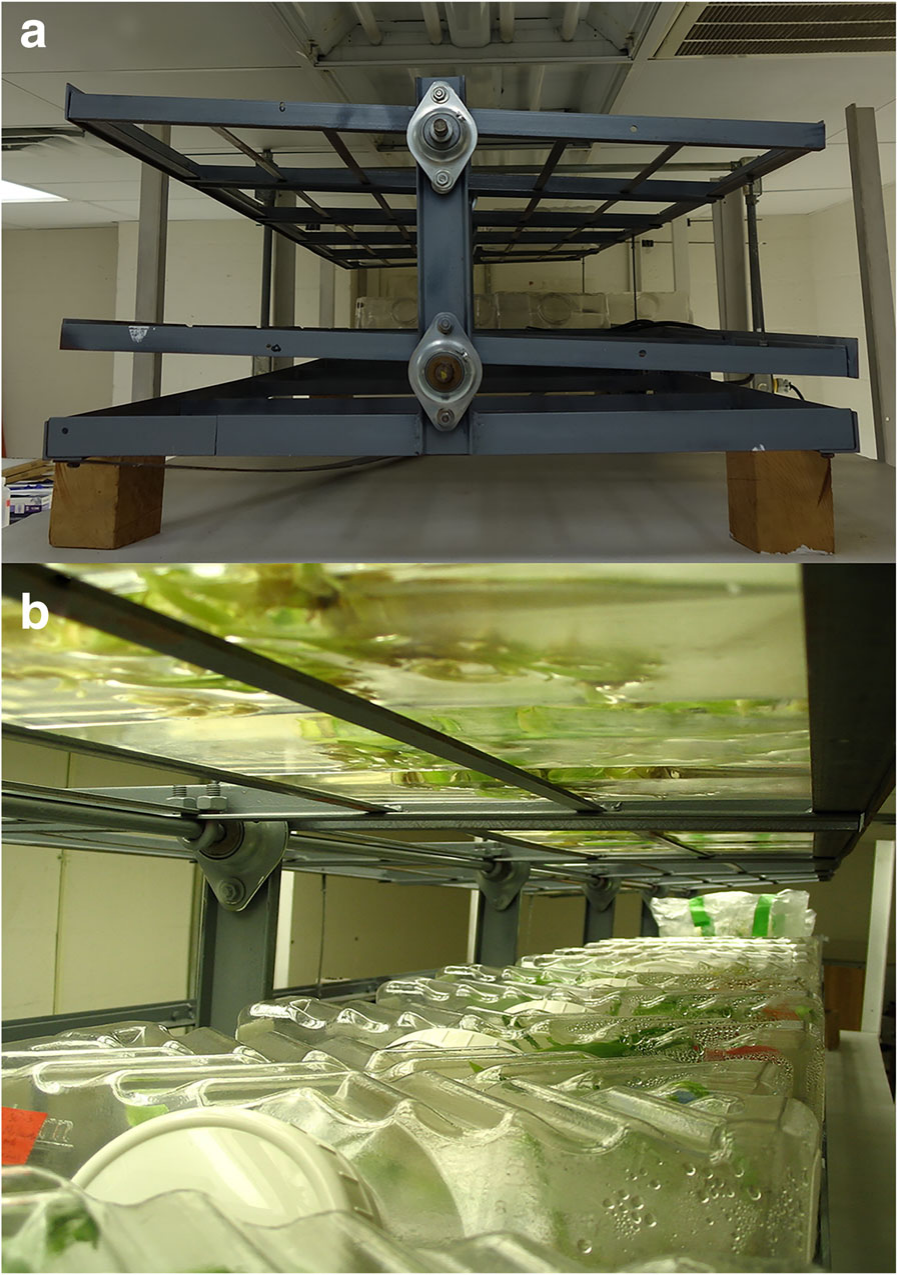
The intermittent immersion rocker system. Periodic wetting and drying of tissue was created when the shelving was gently rocked by the motor (1 rpm) (**a**). The paired shelves were tilted once a minute. Viewing the underside of the vessels on the top shelf (**b**), the shallow film of liquid medium barely covers the plant surface. Plants grow in the 2.5 L gaseous headspace of the vessels as seen on lower shelf

**Fig. 2:**
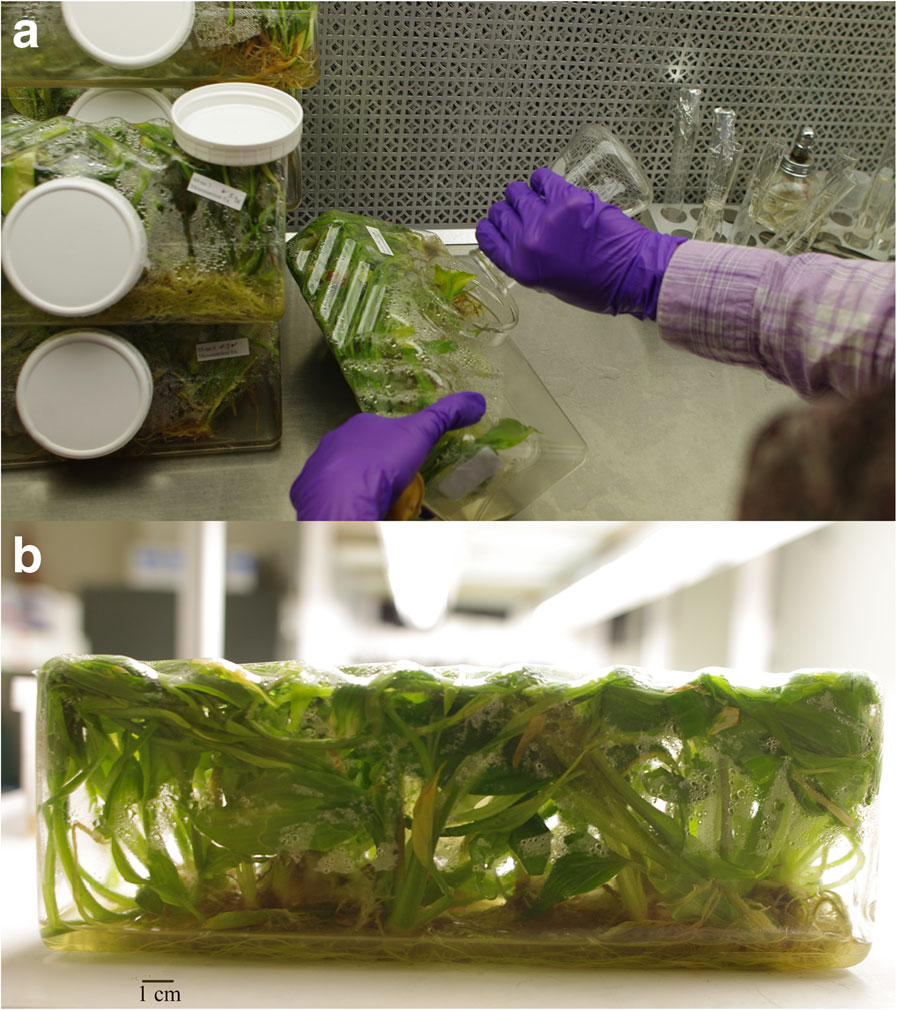
The fed-batch technique of *Curcuma longa* L. in the bioreactor. **a** The fed-batch was applied twice during the 5 month growth period. The volume of water and sucrose concentration was returned to the set point in the sucrose fed-batch subset (SF), additional to that the mineral concentrations were returned to the set point in the mineral-sucrose fed-batch subset (NSF). **b** *Curcuma longa* L. 35–1 filled the 2.5 L bioreactor during 5 months with nutrient supplementation

Turmeric rhizomes were grown on 40 mL modified MS media [21] in cylindrical glass jar (180 mL) with plant density (3, 6, 9 buds/vessel) for 3 cycles of 35 days per cycle then rhizomes were transferred to liquid modified MS media in bioreactor (2.5 L Liquid Lab Vessels, Southern Sun Inc., Hodges, SC, USA) with plant density (6, 12, 18 buds/vessel) [17]. The bioreactors were set on an intermittent immersion rocker system [15, 17, 20] with one rotation per min to allow rhizomes dry and wet in the thin film liquid media (Fig. 1). Liquid MS media [21] was modified with (NH_4_)_2_SO_4_, (5 mM), sucrose (5% m/v), benzyladenine (3 μM) [22] different combinations of in vitro factors (Additional file 1: Table S1), plant density and mineral concentrations, PO_4_^3−^ (1.25, 3.75, 6.25 mM), Ca^2+^ (3, 6, 9 mM), Mg^2+^ (1.5, 3, 4.5 mM), and KNO_3_ (20, 40, 60 mM), were selected by d-optimal criteria [17]. The set of experimental treatments was duplicated with each subset receiving two different fed-batch techniques, sucrose fed-batch (SF) and nutrient-sucrose fed-batch (NSF) applied twice during the 5 months culture (Fig. 2) and previously described by El-hawaz et al. [17]. The control plants were grown on 35 mL liquid MS medium [21] supplemented with 1 μM benzyladenine and 3% *w*/*v* sucrose in a cylindrical glass jar (180 mL) with a Magenta B-cap closure (Magenta Crop, Chicago, IL, USA). Plants were subcultured at 23 ± 2 °C for four 35 days cycles under 25–30 μmol m^− 2^ s^− 1^ photosynthetically active radiant for 16 h per day.

### Greenhouse growth

Three explants transferred from each bioreactor were acclimatized under the mist (latitude = 34.67350, longitude = − 82.83261; 60% shade cloth; mist cycle was 6 s on every 16 min during daylight hours) in Fafard® 2B (Canadian sphagnum peat moss, bark, perlite, vermiculite, wetting agent, and dolomitic limestone; Sun Gro Horticulture, Agawam, MA, USA) without starter fertilizer for SF subset, and with starter fertilizer for NSF subset for 21 days in the summer. In large pots (8 L), SF subset plant was placed in soilless mixture without any fertilizer considered the low-input treatment and NSF subset plant was placed in Fafard® 2B with a complete charge of starter nutrients and considered as the high-input treatment. Pots were irrigated manually once each five days without fertilizer in the low-input treatment, and with 100 ppm N of (15–15-15 4Ca 2 Mg, Jack’s professional LX. J.R. Peters, Inc.) in the high-input treatment [20]. Control plants were irrigated manually once each three days for two months then transferred to the field for three months. Control plants were set in the sun to dry before the harvesting. Harvested turmeric rhizomes were stored at − 80 °C until processing for GC-MS analysis.

### Sample preparation

Rhizomes from treatments and control were frozen immediately after harvest and then samples for analysis were freeze-dried and ground to a fine powder. The turmeric powder from each sample (0.5 g × 3 replicates) was extracted by shaking with 10 mL n-hexane at 40 °C for 30 min. The mixture was centrifuged at 4000 rpm for another 10 min at 4 °C. The clear supernatant was decanted into a conical flask containing about 0.5 g anhydrous sodium sulfate. The extraction procedure was repeated two more times by adding fresh n-hexane (10 mL) to the plant residue in the centrifuge tube. The collected hexane extracts were filtered (0.45 PTFE membrane filter), and evaporated under nitrogen (N_2_) gas until complete dryness. Samples were prepared as 2.5 mg/mL in acetonitrile, and internal standard (oleic acid methyl ester) was added prior to GC-MS.

### GC-MS analysis

Gas chromatography-mass spectrometry (GC-MS) analysis was carried out on an Agilent 7890A GC system attached to an Agilent 5975C MS detector. An Agilent HP-5 ms (30 m × 0.25 mm ID 0.25 μm film thickness) was used for separation of volatile constituents using helium as a carrier gas at a flow rate of 1.2 mL/min. The oven temperature was programed at 60 °C for 5 min, rising to 180 °C at 4 °C/min, then to 280 °C at 15 °C/min with total run time of 60 min. GC-MS parameters at electron impact (EI) mode included ionization voltage 70 eV, injector temperature of 250 °C with injector port set to split mode (1:10) and a mass scan range of 100–800 *m/z*. Identification of compounds was based on comparison of mass spectra with the GC-MS system data bank (NIST 05 library), comparison with published data, and retention indices.

### Measurements

The fresh and dry mass of turmeric rhizomes after 6 months in the greenhouse and the control plants were measured. The concentration of the volatile components in the hexane extract was calculated based on their peak areas in the GC chromatogram in comparison to standard curve constructed with authentic reference curcumenone (Chem Faces, China). Results were expressed as mg curcumenone equivalent/g dried rhizome mass (DM). Percentage of each identified compound was measured based on its peak area relative to total area of the GC chromatogram. Measurements were carried out in triplicate and means were calculated. The concentrations of identified compounds were presented to identify the optimal in vitro formula that induced synthesis and accumulation in turmeric rhizomes. The predicted improvement (fold) was calculated by divide the concentration of each compound in the treatments on the average concentration of the control.

### Statistical analysis and experimental design

The response surface method was used to model responses to five continuous factors at three levels using 32 design points with 3 additional points as true replicates that were selected by d-optimal criteria as described in [17]. The entire experiment was duplicated to apply two levels of the nominal factor (fed-batch technique). The fed-batch treatment in vitro was continued as fertilizer treatment in the greenhouse. The stepwise forward method was used to select the response surface models that presented in tables with the ANOVA summary, *R*^2^, adjusted *R*^2^ (*R*^2^_a_), and F *statistic* at *P*-value. The software JMP version 13.0 (SAS Inst., Cary, NC, USA) was used to make the data analysis and graphs.

## Results

### Qualitative and relative-quantitative analysis of volatile constituents

**Fig. 3:**
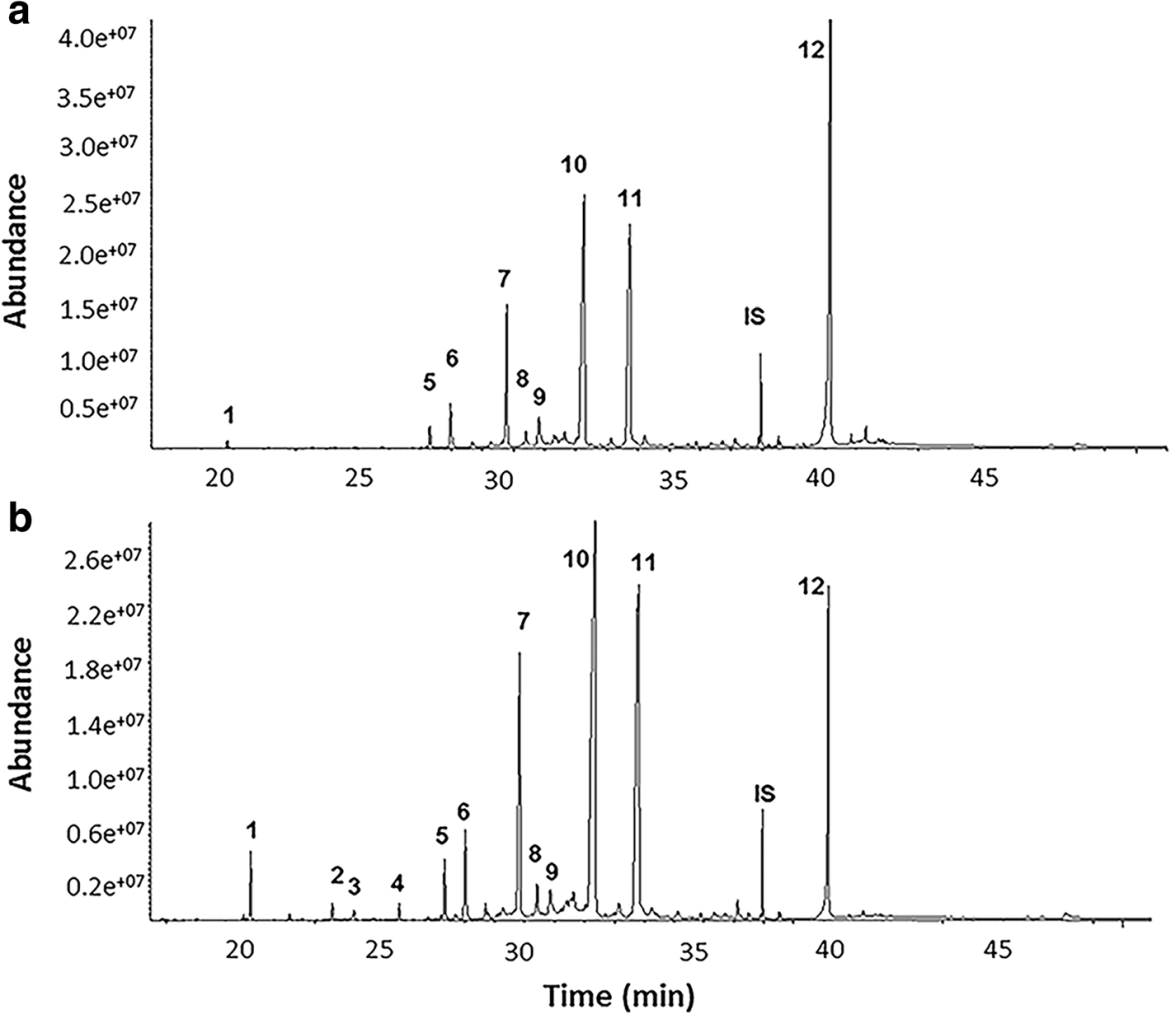
GC-MS Chromatogram of *Curcuma longa* L. hexane extract. Representative GC-MS profile of *Curcuma longa* L. **a** Low-input fertilizer treatment SF 35 and (**b**) High-input fertilizer treatments NSF 35. Identification of peaks (**1**–**12**) was included in Table 1. IS = internal standard

GC-MS analysis of the hexane extract showed that sesquiterpenes represented the major components of the GC-MS chromatogram of *C. longa*, comprising > 65% of total peak area. Identified sesquiterpenes ranged from non-oxygenated, mono-oxygenated and di-oxygenated chemical structures (Table 1). Two isomeric forms of curcumenol, tentatively identified here as curcumenol isomer I (**10**) and curcumenol isomer II (**11**), constituted the major components of the GC chromatogram, accounting for 28.68 ± 0.91% and 17.96 ± 0.69% of the total volatiles identified in the control plants, respectively (Table 1). Isocurcumenol (**6**) and curcumenone (**9**) (*m/z* 234 [M]^+^, C_15_H_22_O_2_) were also identified at lower percentages (3.24 ± 0.14% and 2.10 ± 0.22%, respectively). Other identified compounds included germacrone (**7**), germacrene D (**2**), germacrene A (**3**), germacrene B (**4**), β-elemene (**1**), β-elemenone (**5**), and neocurdione (**8**). Germacrene A (**3**), B (**4**), and D (**2**), were under the limit of quantitation in the low-input fertilizer rhizomes (Fig. 3) and the control. The last eluted peak (40.17 min) was tentatively identified as methenolone (**12**) ([M]^+^ 302, C_20_H_30_O_2_, 20.95 ± 0.49%), a steroidal compound. The percentage of each identified compound in the GC-MS chromatogram based on peak area measurements was included in Table 1.

**Table 1:** Compounds identified in the hexane extract of *Curcuma longa* L. 35–1 rhizomes by GC-MS

	Compound	Rt (min)	Molecular formula	Molecular weight	Control peak area%	Low-input peak area%	High-input peak area%
1	β-elemene	20.83	C_15_H_24_	204	0.85 ± 0.14	0.33 ± 0.04	1.96 ± 0.40
2	Germacrene D	23.57	C_15_H_24_	204	0.16 ± 0.06	0.07 ± 0.01	0.57 ± 0.16
3	Germacrene A	24.3	C_15_H_24_	204	0.14 ± 0.04	0.14 ± 0.2	0.36 ± 0.08
4	Germacrene B	25.81	C_15_H_24_	204	0.12 ± 0.01	0.10 ± 0.02	0.45 ± 0.13
5	β-elemenone	27.33	C_15_H_22_O	218	0.88 ± 0.04	1.04 ± 0.23	1.65 ± 0.14
6	Isocurcumenol	27.97	C_15_H_22_O_2_	234	3.24 ± 0.14	2.78 ± 0.33	3.21 ± 0.20
7	Germacrone	29.81	C_15_H_22_O	218	4.46 ± 1.51	6.14 ± 1.26	9.64 ± 0.72
8	Neocurdione	30.42	C_15_H_24_O_2_	236	1.48 ± 0.10	1.08 ± 0.28	1.42 ± 0.14
9	Curcumenone	30.85	C_15_H_22_O_2_	234	2.10 ± 0.22	3.37 ± 0.73	2.16 ± 0.40
10	Curcumenol isomer I	32.39	C_15_H_22_O_2_	234	28.68 ± 0.9	25.94 ± 2.8	29.56 ± 2.0
11	Curcumenol isomer II	33.75	C_15_H_22_O_2_	234	17.96 ± 0.7	22.56 ± 3.2	21.82 ± 2.9
12	Methenolone	40.14	C_20_H_30_O_2_	302	20.95 ± 0.5	19.36 ± 5.3	8.56 ± 2.75
	Total				81.01 ± 1.4	82.93 ± 3.6	81.36 ± 3.6

The volatile content in turmeric rhizomes of the control and the fertilizer treatments using *GC-MS* analysis. Percentages were measured based on peak area of each compound to total peak area of the *GC* chromatogram ± standard deviation (*n* = 3)

### Volatiles detected in all fertilizer treatments

**Fig. 4:**
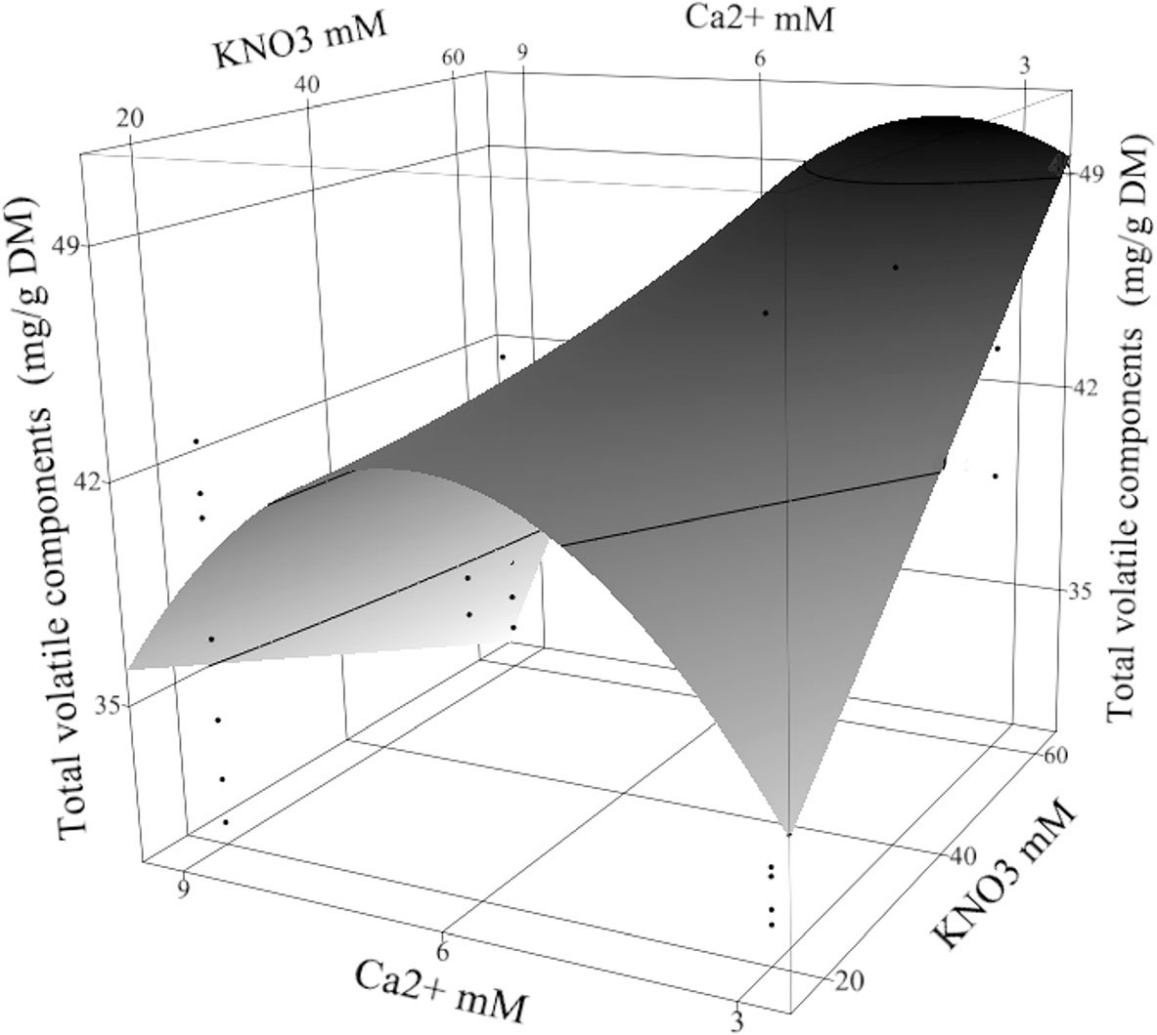
Total content of volatiles under the high-input fertilizer treatment. Total volatile content (mg/g DM) in high-input fertilized rhizome of *Curcuma longa* L. 35–1 was shown under the effect of in vitro Ca^2+^ × KNO_3_ mM interaction. The contour surface and surface plus residual data points appeared on the response surface plot

The total content of volatile compounds determined in the nutrient treated rhizome was significantly affected by fertilizer treatments in the greenhouse, in vitro concentration of Ca^2+^, and the interaction of Ca^2+^ and KNO_3_ (Table 2). The content was higher in the high-input fertilizer (49.7 ± 9 mg/g DM) than the low-input fertilizer plants (26.6 ± 9 mg/g DM) grown on in vitro media contained 4 mM Ca^2+^, 60 mM KNO_3_, and 5 mM NH_4_^+^ (Fig. 4) that suggested the more fertilizer in the greenhouse produced higher concentration volatile components in rhizomes. Both fertilizer treatments had greater concentrations of volatiles than the control (15.28 ± 2.68 mg/g DM) grown on MS medium contained 3 mM Ca^2+^, 20 mM K^+^, 39 mM NO_3_^−^, and 20 mM NH_4_^+^ [21]. The model terms in Table 2 shows the Ca^2+^ (3–9 mM) had a negative effect on the volatile content but the optimal level of Ca^2+^ was higher than the lowest level (3 mM). Interpretation of Ca^2+^ main effect was misleading because Ca^2+^ interacted significantly with KNO_3_ and must be qualified depending on KNO_3_ impact (Fig. 4). At 60 mM KNO_3_, the volatile content was maximized with little Ca^2+^ (4 mM). Reduced KNO_3_ to 20 mM required more Ca^2+^ (6 mM) to increase the content of total volatiles in turmeric rhizome. The strength of the response surface model (RSM) over single factor experiments is its sensitivity to many second order effects. The maximum increase in volatile compounds was predicted from RSM model (similar to those in Table 2) derived from d-optimal selection of 35 treatment points from 243 experimental points in the design space. The predicted improvement of total volatile compounds in the high-input fertilizer rhizome was 6.5 ± 1.2 fold and in the low-input fertilizer rhizome was 3.5 ± 1.2 fold comparing to the control content.

**Table 2:** The significant terms of the response surface models of GC-MS analysis in *Curcuma longa* L.35–1 rhizome

	Compounds (^a^)									
Significant model terms	(1)	(5)	(6)	(7)	(8)	(9)	(10)	(11)	(12)	Total
High-input Fertilizer	0.0001	0.0001	0.0001	0.0001	0.0001	0.0001	0.0001	0.0001		0.000.0001
Ca^2+^ mM	0.0267 0.0101	0.0013	0.0059	0.0019	0.0478	0.0001	0.0042	0.0121	0.0395	0.0166
KNO_3_ mM						0.0079	0.0200			
Ca^2+^ × KNO_3_ mM		0.0312	0.0333	0.0104		0.0070	0.001	0.0076		0.0458
Mg^2+^ mM						0.0073				
Mg^2+^ × KNO_3_ mM						0.0003			0.0208	
Ca^2+^ × Mg^2+^ mM							0.013			
High-input × Buds/Vessel					0.0416					
High-input × PO_4_^3−^ mM						0.0083			0.0186	
High-input × Ca^2+^ mM					0.0422				0.0340	
High-input × KNO_3_ mM					0.0494					
Buds/Vessel× PO_4_^3−^ mM						0.0378				
(PO_4_^3−^ mM)^2^					0.0041	0.0060			0.0324	
(Ca^2+^ mM)^2^										
*R*^*2*^ =	0.804	0.785	0.687	0.785	0.772	0.688	0.771	0.701	0.448	0.660.666

^a^(1) *β*-elemene, (5) *β*-elemenone, (6) isocurcumenol, (7) germacrone, (8) neocurdione, (9) curcumenone, (10) curcumenol isomer *I*, (11) curcumenol isomer *II*, and (12) methenolone

Curcumenol isomers I (**10**) and II (**11**) were the major sesquiterpenes in MS control turmeric rhizomes (Table 1). In the nutrient treatments, the high-input fertilizer treatments had the most significant effect followed by the interaction of Ca^2+^ and KNO_3_ (Table 2) on the content of curcumenol isomer I (**10**) and curcumenol isomer II (**11**). In the high-input fertilizer, the content of curcumenol isomer I (**10**) was the greatest at 28.05 ± 3.14 mg/g DM, and 16.48 ± 3.14 mg/g DM with the low-input fertilizer when the in vitro factors were 6.25 mM PO_4_^3−^, 3 mM Ca^2+^, 1.5 mM Mg^2+^, and 60 mM KNO_3_. The control rhizome had low content of curcumenol isomers I (**10**) (5.49 ± 0.81 mg/g DM). The predicted improvement of curcumenol isomer I (**10**) was calculated similar to the total volatile components where the increase of curcumenol isomer I (**10**) in the high-input fertilizer rhizome was 10.2 ± 1.1 fold and in the low-input fertilizer rhizome was 6.0 ± 1.1 fold comparing to the control content.

**Fig. 5:**
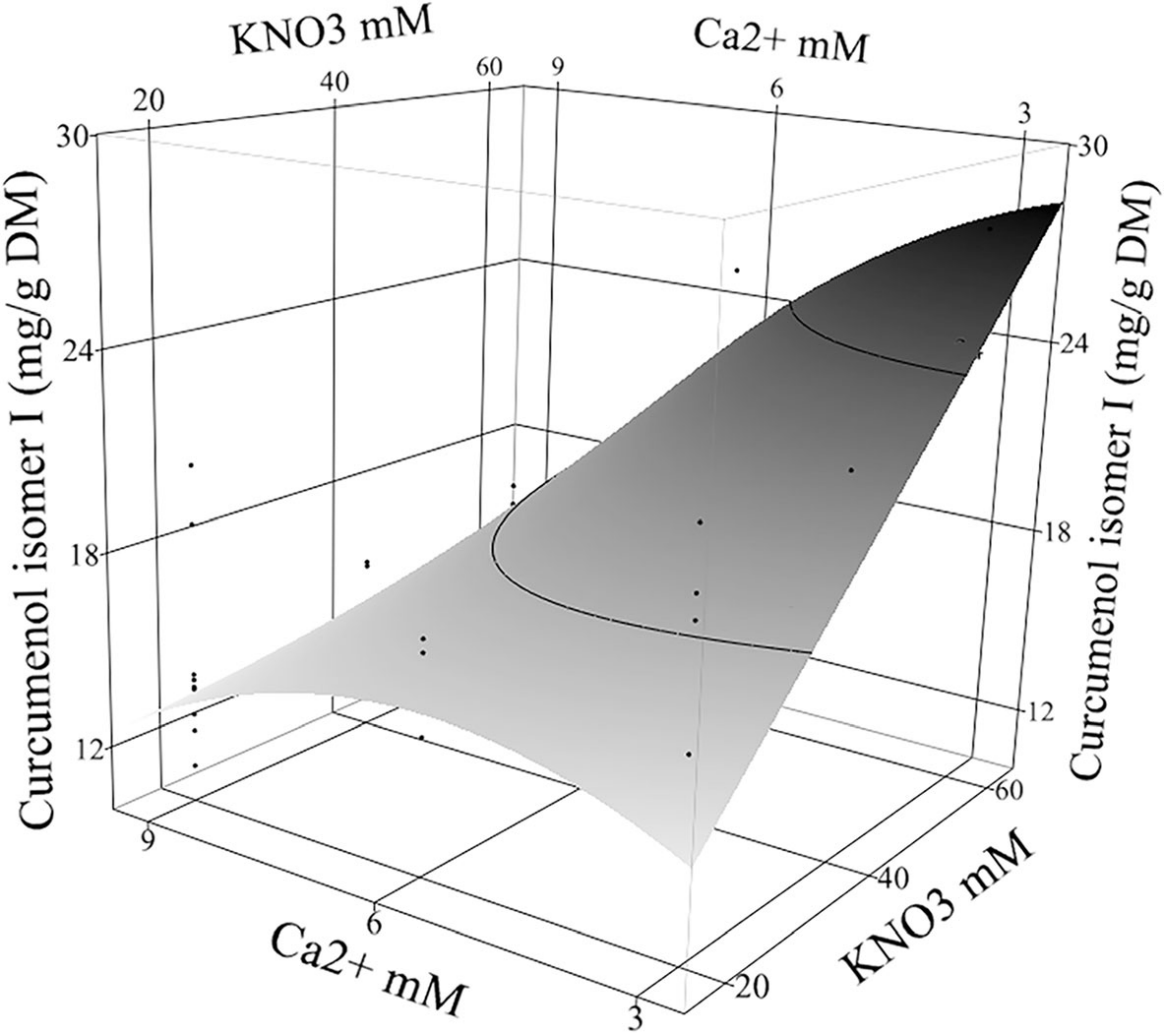
Curcumenol isomer I content under the high-input fertilizer treatment. Curcumenol isomer I content (mg/g DM) in high-input fertilized rhizome of *Curcuma longa* L. 35–1 was shown under the effect of in vitro Ca^2+^ × KNO_3_ mM interaction when the other factors were set as 6.25 mM PO_4_^3−^ and 1.5 mM Mg^2+^. The contour surface and surface plus residual data points appeared on the response surface plot

Curcumenol isomer II (**11**) content was the greatest at 16.84 ± 2.53 mg/g DM of high-input fertilizer, and 9.96 ± 2.53 mg/g DM with the low-input fertilizer when the in vitro factors set as 3 mM Ca^2+^, 1.5 mM Mg^2+^, and 60 mM KNO_3_. However, the control rhizome content of curcumenol isomer II (**11**) was only 3.77 ± 0.74 mg/g DM. Increasing Ca^2+^ concentration (> 3 mM) interacted with 60 mM KNO_3_ to reduce curcumenol isomers I (**10**) and II (**11**) contents (Fig. 5). At 60 mM KNO_3_, the lowest Ca^2+^ concentration (3 mM) is increased curcumenol isomers I (**10**) and II (**11**) contents. The predicted improvement of curcumenol isomer II (**11**) was calculated similar to the total volatile components where the increase of curcumenol isomer II (**11**) in the dry high-input fertilizer rhizome was 13.9 ± 1.5 fold and in the low-input fertilizer rhizome was 11.3 ± 1.5 fold comparing to the control content.

**Fig. 6:**
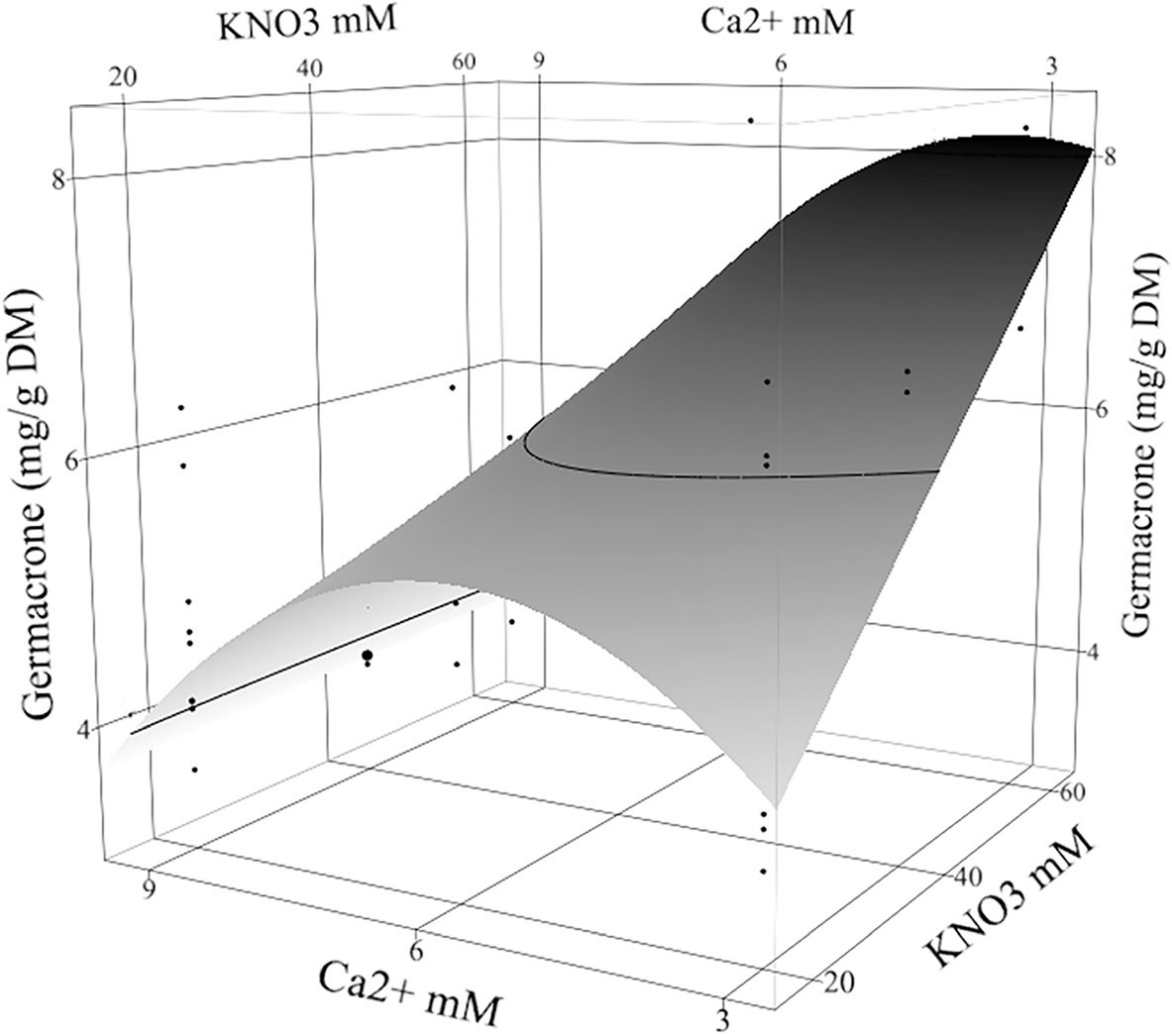
Germacrone content under the high-input fertilizer treatment. Germacrone content (mg/g DM) in high-input fertilized rhizome of *Curcuma longa* L. 35–1 was shown under the effect of in vitro Ca^2+^ × KNO_3_ mM interaction when Mg^2+^ set as 1.5 mM. The contour surface and surface plus residual data points appeared on the response surface plot

High fertilizer treatments and reducing the divalent cation Ca^2+^ resulted in significant increases in the accumulation of germacrone (**7**), isocurcumenol (**6**), β-elemenone (**5**), and β-elemene (**1**) followed by the interaction effect of Ca^2+^ with KNO_3_ (Table 2). In the high-input fertilizer treatment, the content of germacrone (**7**) was maximized to 8.21 ± 1.07 mg/g DM with set of 3.5 mM Ca^2+^, 1.5 mM Mg^2+^, and 60 mM KNO_3_ (Fig. 6) and the content under the low-input fertilizer treatment (4.59 ± 1.07 mg/g DM) was higher than the control (1.05 ± 0.10 mg/g DM). The predicted improvement of germacrone (**7**) was calculated similar to the total volatile components where the increased fold of germacrone (**7**) in high-input fertilizer was 15.6 ± 2.0 fold and in the low-input fertilizer was 8.7 ± 2.0 fold comparing to the control content. The content of isocurcumenol (**6**) was the greatest at 2.02 ± 0.38 mg/g DM with the high-input fertilizer and 1.04 ± 0.38 mg/g DM with the low-input fertilizer when the in vitro factors set as 4.3 mM Ca^2+^ and 60 mM KNO_3_. However, the control rhizome had 0.62 ± 0.10 mg/g DM. The predicted improvement of isocurcumenol (**6**) was calculated similar to the total volatile components where the increase of isocurcumenol (**6**) in high-input fertilizer was 6.6 ± 1.2 fold and in the low-input fertilizer was 3.4 ± 1.2 fold comparing to the control content. The content of β-elemenone (**5**) in the rhizome was the greatest at 1.08 ± 0.17 mg/g DM with the high-input fertilizer when the in vitro factors set as 18 buds/vessel, 4.5 mM Ca^2+^, and 60 mM KNO_3_. In the low-input fertilizer, rhizome content (0.41 ± 0.17 mg/g DM) was higher than the control (0.15 ± 0.03 mg/g DM). The predicted improvement of β-elemenone (**5**) was calculated similar to the total volatile components where β-elemenone (**5**) increased in the high-input fertilizer to 14.0 ± 2.2 fold and in the low-input fertilizer to 5.3 ± 2.2 fold comparing to the control content. β-elemene (**1**) content was the greatest at 1.46 ± 0.25 mg/g DM with the high-input fertilizer and 0.44 ± 0.25 mg/g DM with the low-input fertilizer when the in vitro factors set as 18 buds/vessel, 6.25 mM PO_4_^3−^, and 5.8 mM Ca^2+^. The control rhizome content was 0.11 ± 0.0.5 mg/g DM. The predicted improvement of β-elemene (**1**) was calculated similar to the total volatile components where the increase of β-elemene (**1**) in the high-input fertilizer rhizome was 26.5 ± 4.7 fold and in the low-input fertilizer rhizome was 8.0 ± 4.7 fold comparing to the control content.

The highest levels of KNO_3_ (60 mM) paired with Ca^2+^ concentration ranges from 3.5 to 5.8 mM maximized the contents of germacrone (**7**), isocurcumenol (**6**), β-elemenone (**5**), and β-elemene (**1**) in the greenhouse rhizomes. The concentrations of Ca^2+^, K^+^, and NO_3_^−^ were higher than standard MS medium (3, 19 and 40 mM, respectively; [22]) with NH_4_^+^ was set at 5 mM in our experimental medium (compared to 20 mM in MS medium). At 20 mM KNO_3_, 6 mM of Ca^2+^ was required to increase the contents of the compounds in turmeric rhizomes. At 9 mM Ca^2+^, there was no significant difference between KNO_3_ concentrations (Fig. 6).

Neocurdione (**8**) content was significantly affected by the fertilizer treatments, the in vitro nutrient factors PO_4_^3−^, Ca^2+^, KNO_3_, and plant density (Table 2). With the high-input fertilizer treatments, the content of neocurdione (**8**) was maximized to 0.77 ± 0.15 mg/g DM and (0.34 ± 0.15 mg/g DM at the low-input fertilizer treatment) when the in vitro factors were set as 18 buds/vessel, 1.25 mM PO_4_^3−^, 3 mM Ca^2+^, and 20 mM KNO_3_. The control rhizome had lower content than the fertilizer treatments (0.23 ± 0.03 mg/g DM). The predicted improvement of neocurdione (**8**) was calculated similar to the total volatile components where the increase of neocurdione (**8**) in the high-input fertilized was 6.6 ± 1.3 fold and in the low-input fertilized was 2.9 ± 1.3 fold comparing to the control content. High-input fertilizer increased the accumulation of neocurdione (**8**) with significant effect of high in vitro plant density (18 bud/vessel), but all mineral concentrations in the micropropagation media had no significant effect on neocurdione (**8**) content. At the low-input fertilizer, mineral concentration significantly affected the content where rhizome’s neocurdione (**8**) increased with low PO_4_^3−^, Ca^2+^, and KNO_3_ at any plant density. The accumulation of neocurdione (**8**) in the greenhouse rhizome required limited in vitro nutrient with increasing the initial plant density.

**Fig. 7:**
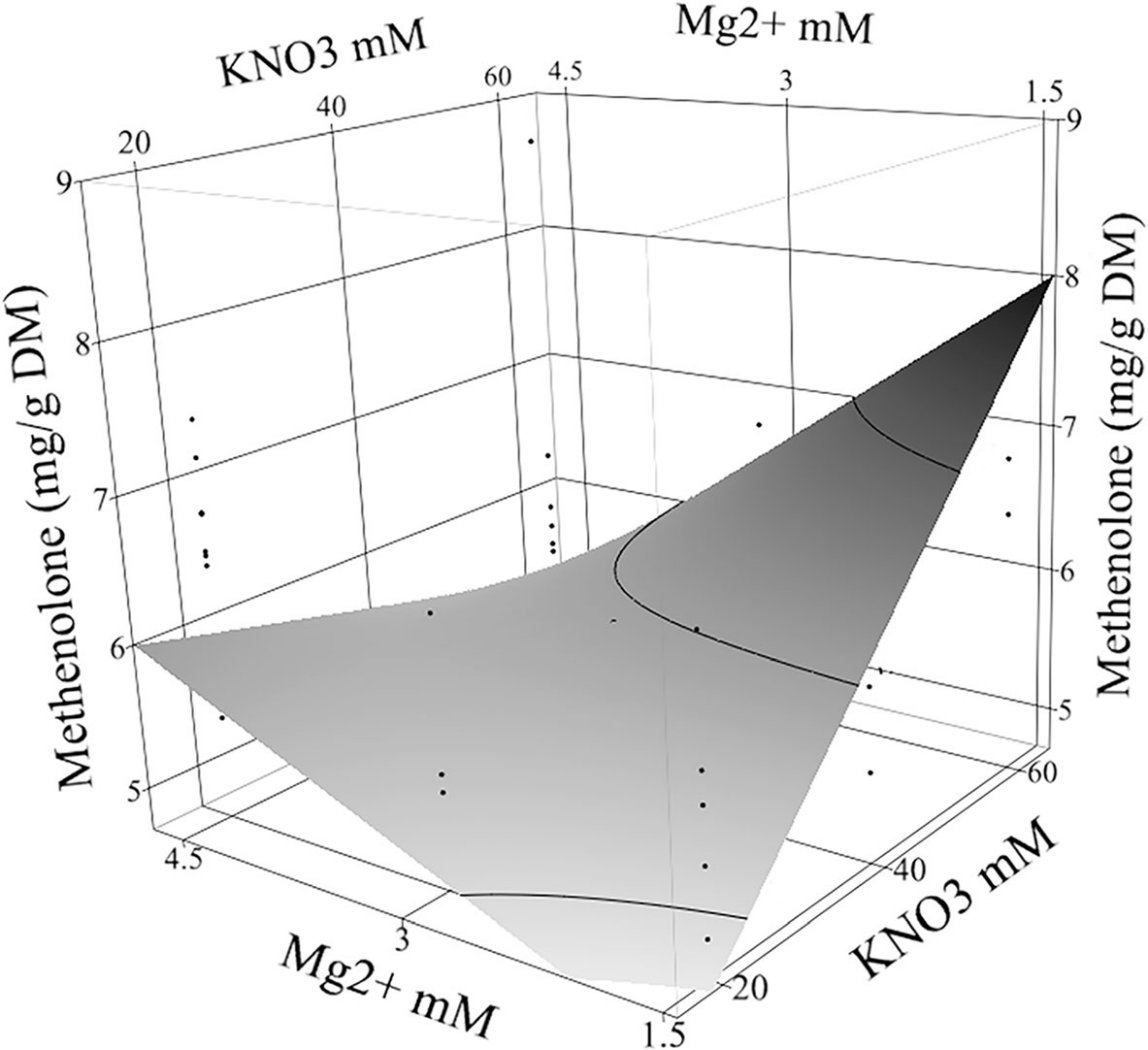
Methenolone content under the high-input fertilizer treatment. Methenolone content (mg/g DM) in low-input fertilized rhizome was shown under the effect of in vitro Mg^2+^ × KNO_3_ mM interaction when other factors set as 6.25 mM PO_4_^3−^, and 6.2 mM Ca^2+^. The contour surface and surface plus residual data points appeared on the response surface plot

The steroid-methenolone (**12**) content was affected by the interaction of fertilizer treatments with PO_4_^3−^ and Ca^2+^, and the interaction of Mg^2+^ and KNO_3_ (Table 2). In the high-input fertilizer treatment, the content reached a maximum of 8.17 ± 1.5 mg/g DM when in vitro factors were 6.25 mM PO_4_^3−^, 6.2 mM Ca^2+^, 1.5 mM Mg^2+^, and 60 mM KNO_3_ (Fig. 7). At high KNO_3_ (60 mM), the low Mg^2+^ (1.5 mM) promoted the accumulation of methenolone (**12**) in tumeric rhizomes. Increase PO_4_^3−^ increased methenolone (**12**) content. However, under the low-input fertilizer, lower PO_4_^3−^ increased the content. At 20 mM KNO_3_, the high Mg^2+^ (4.5 mM) promoted the accumulation of methenolone (**12**) to 7.04 ± 1.5 mg/g DM with 1.25 mM PO_4_^3−^, 5.12 mM Ca^2+^, 4.5 mM Mg^2+^, and 20 mM KNO_3_. The control rhizome had lower content (3.60 ± 0.49 mg/g DM) than treatment rhizomes. The predicted improvement of methenolone (**12**) was calculated similar to the total volatile components where methenolone (**12**) increased in the high-input fertilizer rhizome to 4.5 ± 0.8 fold and in the low-input fertilizer rhizome to 3.0 ± 0.8 fold comparing to the control content.

Curcumenone (**9**) content was significantly influenced by many factors in this experiment including the fertilizer treatment, Ca^2+^, KNO_3_ and its interaction with the divalent cations Ca^2+^ and Mg^2+^, PO_4_^3−^ and its interaction with the fertilizer treatments, and plant density (Table 2). In both fertilizer treatments, the concentration reached a maximum of 2.5 ± 0.24 mg/g DM with in vitro factors 18 buds/vessel, 1.25 mM PO_4_^3−^, 3 mM Ca^2+^, 1.5 mM Mg^2+^, and 60 mM KNO_3_ and that was greater than the control contents (0.25 ± 0.13 mg/g DM). The predicted improvement of curcumenone (**9**) was calculated similar to the total volatile components where curcumenone (**9**) increased in the high-input fertilized rhizome to 22.9 ± 1.8 fold and in the low-input fertilized rhizome to 20.4 ± 1.8 fold comparing to the control content. At 60 mM KNO_3_, the concentration of curcumenone (**9**) increased with decreasing Mg^2+^ to 1.5 mM. At 20 mM KNO_3_, Mg^2+^ had no significant effect on the content. Low PO_4_^3−^ (1.25 mM) increased curcumenone (**9**) accumulation with increasing plant density that reduced significantly the fertilizer treatments’ effect. Increasing PO_4_^3−^ induced curcumenone (**9**) accumulation with the low plant density and high-input fertilizer.

### Compounds detected in the high-input fertilizer treatment

Germacrenes A (**3**), B (**4**), and D (**2**) were detected in the high-input fertilizer treatment but they were under the detection level in both low-input fertilizer treatment (Fig. 3) and the control rhizomes. In the high-input fertilizer treatment, Ca^2+^ affected significantly the content of germacrene D (**2**). The optimal level of Ca^2+^ (6 mM) maximized germacrene D (**2**) content to 0.38 ± 0.15 mg/g DM when combined with the highest concentrations of KNO_3_ (60 mM) and Mg^2+^ (4.5 mM). The models of germacrene A (**3**) and germacrene B (**4**) were not significant and in vitro treatments had no effect on their concentrations.

### Association among multiple compound responses

In the low-input fertilizer treatment, a significant correlation (*r* = 0.9) was found among the different sesquiterpene compounds in turmeric rhizome extract and sesquiterpene compounds significantly correlated (*r* = 0.7) with plant growth in the greenhouse (fresh and dry mass of leaves, rhizomes, and roots) as well in the control rhizomes. The volatile components were well correlated when the plant growth when was limited by nutrient input. On the other hand, volatile components concentrations in the high-input fertilizer treatment showed no significant correlation with plant growth in the greenhouse. An over abundance of nutrients caused overgrowth of leaves, rhizomes and roots that were not as efficient in producing the volatile components. The steroid methenolone (**12**) correlated only with curcumenone (**9**) (*r* = 0.6), but not plant growth.

Germacrenes A (**3**), B (**4**), and D (**2**) (Table 1) were under limited detection in the control and low-input treatment and only were quantified in the high input treatments. Concentrations of the minor compounds, germacrenes, in the high-input fertilizer rhizomes were significantly correlated (*r* = 0.9) with other sesquiterpenes, and (*r* = 0.5) with curcumenone (**9**). The overabundance of nutrient caused the production of germacrenes that were well correlated with the production of other sesquiterpenes.

Curcuminoids are the most important class of secondary compounds in turmeric. The content of volatile compounds correlated poorly with the curcuminoid compounds (*r* = 0.3) analyzed in a prior work [19]. Volatile compounds in turmeric rhizome extract likely have divergent pathways than curcuminoids compounds. Comparing the average percentage of each sesquiterpene compound in the control, low and high-input fertilizer treatments showed very strong correlation (r = 0.9) and when steroid compound (methenolone) was pulled out the correlation increased to (*r* = 0.98) showing that *Curcuma longa* L. 35–1 clone profile of sesquiterpenes similar to Table 1.

### Volatile components were affected by fertility and the primary metabolism

**Fig. 8:**
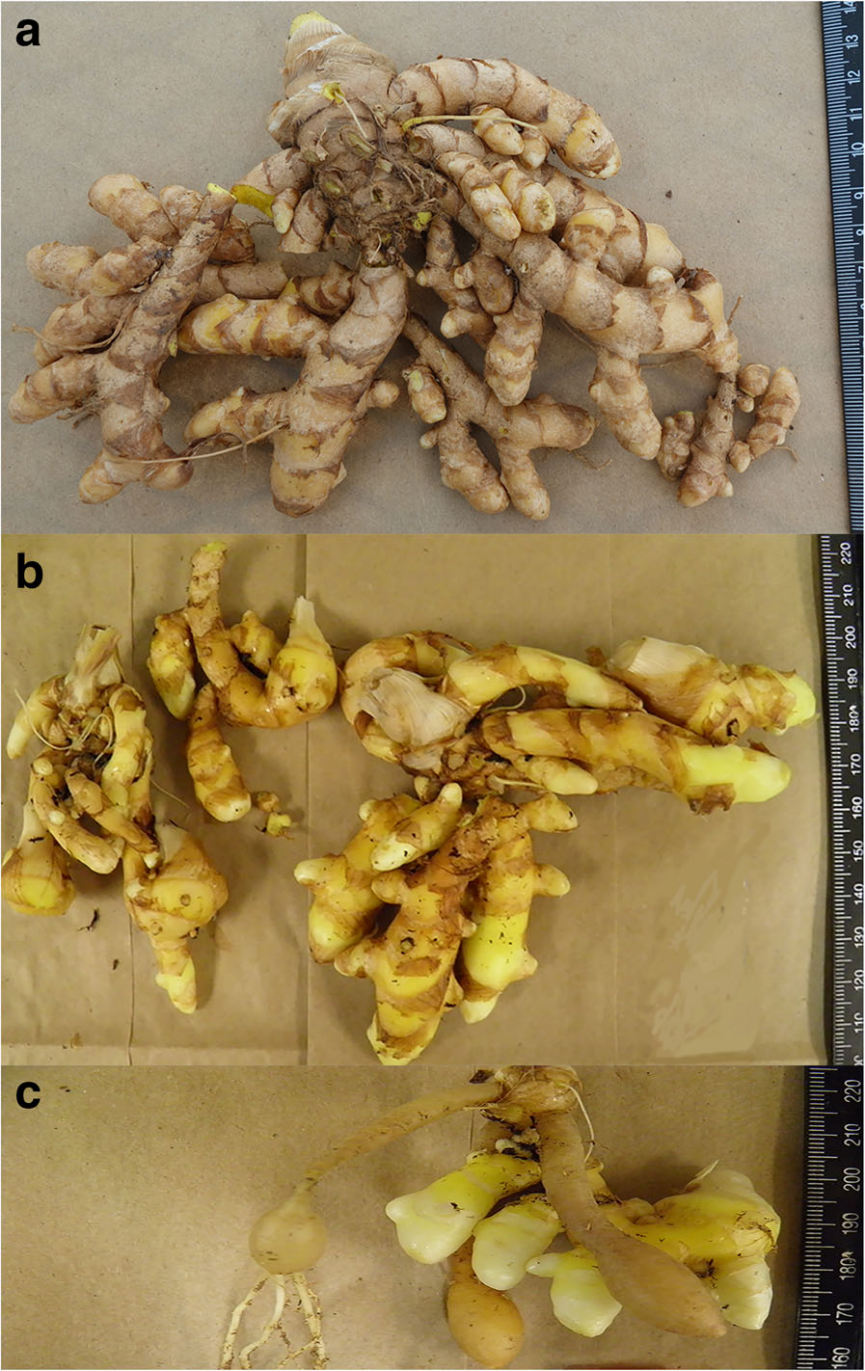
*Curcuma longa* L. rhizomes. The harvested rhizome of *Curcuma longa* L. 35–1 (**a**) control, **b** high-input, and (**c**) low-input fertilizer treatments (experimental point 45) with in vitro factors set as 18 buds/vessel, 6.25 mM PO_4_^3−^, 3 mM Ca^2+^, 1.5 mM Mg^2+^, and 60 mM KNO_3_

Considering the dry rhizome mass in both fertilizer treatments, the amount of the volatile compounds was greater in the high-input than the low-input fertilizer. The d-optimal design selection criteria used to produce these results reduced the number of tested points from 243 to 35 points, and one tested point (treatment 45) closest to the optimal was shown as an example (Fig. 8). The amount of volatiles produced was compared by multiplying the dry mass of rhizome by the concentration in the high-input / low-input fertilizer listed for each compound: 23.78/0.04 mg β-elemene, 16.60/0.29 mg β-elemenone, 32.10/1.06 mg isocurcumenol, 95.82/1.96 mg germacrone, 9.29/0.30 mg neocurdione, 13.92/1.17 mg curcumenone, 291.71/9.95 mg curcumenol isomer I, 224.63/9.07 mg curcumenol isomer II, and 44.19/5.20 mg methenolone. The greater mass and concentration conferred in the high fertility treatment produced these overwhelming large differences. Primary metabolism (plant growth) and the production of secondary metabolites were simultaneously enhanced in these systems.

## Discussion

A number of isomeric forms of sesquiterpenes, with the molecular formula C_15_H_22_O_2_, *m/z* 234 [M]^+^, including curcumenol, epicurcumenol, isocurcumenol, procurcumenol and isoprocurcumenol, were previously identified in *C. longa* through isolation, NMR analysis, and other chemical and spectral determination methods [23, 24]. Germacrene A (**3**), B (**4**), and D (**2**) compounds are intermediate compounds in the biosynthesis metabolism [25] that might be caused the well correlation with other sesquiterpene concentrations in the high-input fertilizer treatment rhizome. Germacrene A is bound to sesquiterpene cyclase enzyme [26]. In our results they were found under detection level in the low-input fertilizer treatment and the control rhizomes that might due to their instability [25]. The steroid methenolone was previously identified in *C. aeruginosa* Roxb rhizome [27], and in the volatiles of *Zingiber nimmonii* J. Graham Dalzell [28], *Alpinia purpurata* Vieill [29], and. *Equisetum giganteum* L. [30].

Bisabolane-type sesquiterpenes were one of the main volatile components constituents of *Curcuma* species with ar-turmerone as the major compound [4]. Turmerones are known as the major sesquiterpenes in the volatile components of turmeric rhizome and the content varies from 40 to 60% [11]. The lack of the entire bisabolane-type sesquiterpenes, ar-turmerone, turmerone, curcumene, zingiberene, β-sesquiphellandrene, curlone, β-bisabolene, from our samples of *Curcuma longa* L. 35–1 profile (Table 1) may be due to the variation between genotypes. Genotypes had different profiles of volatile components and GC-MS distinguished two types of *C. longa*, the “yellow type” which contained mainly ar-tumerone, turmerone, culone, zingiberene, 2-carene, and β- sesquiphellandrene, while the “red type” contained carvacrol, citral, methyleugenol, geraniol, menthol and caryophyllene oxide [31]. The clone in this experiment was not like either the “yellow” or “red” types.

The results showed how the clonal profile was impacted by the mineral nutrient and environment. The association among the volatile compounds in this genotypic profile was caused by the interaction of KNO_3_ and Ca^2+^ that had the most significant effect following fertilizer treatments on the accumulation of curcumenol isomers I and II, germacrone, isocurcumenol, and β-elemenone in turmeric rhizome. A similar interaction between Ca^2+^ and KNO_3_ increased the in vitro gain of turmeric fresh mass and plants with greater fresh mass were transferred to the greenhouse with 60 mM KNO_3_ and 3 mM Ca^2+^ [17]. This differs from the MS 1962 medium reported by all other groups working with turmeric [32] that contains 40 mM K^+^, 39 mM NO_3_^−^, 20 mM NH_4_^+^, 3 mM Ca^2+^, 1.5 mM Mg^2+^, and 1.25 mM PO_4_^3−^ [21], and MS was the medium we used for the control plants in this work. The invigoration of plant growth using the altered medium was coincident with the enhanced levels of sesquiterpenes. Studying nitrogen alone suggested a reduction in terpenoids and volatile content with increasing nitrogen supplement in the annual herbal plants [33] and *Juniperus horizontalis* [34]. Calcium involved in enzyme signal transductions in the cytosol [35] that might have a role in sesquiterpenes biosynthesis, especially as they are synthesized in the cytosol from the mevalonate pathway [36]. Calcium has been found regulate the antioxidant metabolism and water relations under the abiotic stress [37, 38] and decreased hydrogen peroxide and thiobarbutric acid [37, 39, 40]. Calcium was involved as a messenger in number of signal transduction pathways, although the calcium binding protein may interact directly with enzymes like sesquiterpene cyclase the most significant enzyme for sesquiterpene metabolism in plants [41]. The increases in the Ca^2+^ level in the nutrient solution increased the volatile components and sesquiterpene content in herbal plants, lemon grass, and bergamot and Japanese mint [42], and *Chrysanthemum coronarium* [43]. In tissue culture, Ca^2+^ enhanced the sesquiterpene production in *Hyoscyamus muticus* root [44]. In the field, applying Ca^2+^ increased carvacrol, γ-terpinene and β-bisabolene in *Satureja hortensis* [45], sesquiterpenes especially germacrene D in *Chrysanthemum boreale* [46].

However, the interaction of KNO_3_ and Mg^2+^ affected the content of curcumenone and methenolone. That might suggest an important role of the balance of the divalent cations Ca^2+^ and Mg^2+^ in the biosynthesis of the volatile components. The optimal concentrations of K^+^ and NO_3_^−^ in vitro were greater than concentrations specify in MS medium (20 and 40 mM respectively). The optimal K^+^ was 3 to 4× and the optimal NO_3_^−^ was 1.5× greater than MS medium. However, previous work indicated KNO_3_ concentration was more important than Ca^2+^ and Mg^2+^ for turmeric growth, both in vitro and greenhouse growth [17, 47].

In the optimal growth media [20], PO_4_^3−^ was in the highest concentration (6.25 mM) and that was related to increasing the supplement of sucrose during in vitro stage [17], which increased rhizome dry biomass in vitro [15]. Under the low-input fertilizer, methenolone concentration increased with 1.25 mM PO_4_^3−^ as well the concentration of curcumenone under both fertilizer treatments, which explained the low correlation between these compounds and growth. The reduction in PO_4_^3−^ from 1.5 to 0.5 mM reduced the growth rate of *Hyoscyamus muticus* root and increased sesquiterpene yield in the bioreactors [48]. [PO_4_^3−^] and [Mg^2+^] significantly affected the content of methenolone and curcumenone (Table 2) and that might cause the high correlation between these compounds. Mineral nutrients play a role in determining the concentration of secondary metabolites in plant where the content of volatile fraction in *Achillea millefolium* leaves altered based on elements availability in the hydroponic system [49].

The optimal contents of the volatile constituents in tumeric rhizome required the same mineral nutrient in vitro as the optimal growth, but we found that curcuminoids required different concentration of minerals to maximize the content. From our results, turmeric growth maximized under the high-input fertilizer treatments with 3.5–4 mM PO_4_^3−^, 5.7–6 mM Ca^2+^, and 60 mM KNO_3_ in vitro mineral nutrients that produced the greatest rhizome fresh mass [20]. However, the highest curcumin concentration was induced under the low-input fertilizer treatments with 6.25 mM PO_4_^3−^, 3 mM Ca^2+^, and 60 mM KNO_3_ in vitro mineral nutrients [19]. Understanding the effect of mineral nutrition would allow determining the adequate supply of mineral in the micropropagation and the greenhouse systems to maximize the secondary metabolites production.

## Conclusion

The content of the volatiles was impacted by prior in vitro treatments as well as by the amount of fertilizer applied during the greenhouse growth. The fertilizer treatments during the greenhouse growth increased the content of the volatiles in the rhizomes. High mineral concentration media with low ammonium concentration (5 mM) were developed to increase the phytochemical production in turmeric rhizomes. The interaction of KNO_3_ and the divalent cation, Ca^2+^, play an important role in accumulation of sesquiterpenes in tumeric rhizome. The interaction of Ca^2+^ and KNO_3_ was similar in both fertilizer treatments in the greenhouse. At the highest KNO_3_ concentration in vitro, increasing Ca^2+^ concentration in vitro increased the total content germacrone (**7**), isocurcumenol (**6**), and β-elemenone (**5**) in turmeric rhizome, but the lowest concentration of Ca^2+^ increased curcumenol isomers I (**10**) and II (**11**), and curcumenone (**9**). The biochemical role of Ca^2+^ in the volatile compounds biosynthesis is still unclear and further investigations are needed. The increase of volatile content in turmeric rhizome can be done without diminishing curcuminoid production. The total volatile content was correlated to the primary metabolism, growth; which is dissimilar to curcuminoid accumulation in rhizomes. Phytochemical concentrations were multiplied significantly by primary metabolism in the ex vitro growth of the rhizome during greenhouse culture. The multifactor design, response surface method (RSM), elucidated important mineral factors that affected the subsequent accumulation of secondary metabolites 6 months after applying the treatments. The multi-factor approach defined (1) the unique chemo types for *C. longa* L. 35–1 and (2) the association between volatile and primary metabolism.

## Additional file

Additional file 1: Turmeric volatile constituents data. (XLSX 26 kb)

## Abbreviations

DM: The dry mass of rhizome
GC-MS: Gas chromatography-mass spectrometry
MS: Murashige and Skoog medium
RSM: Response surface method
