# Correction to: In vitro mineral nutrition of *Curcuma longa* L. affects production of volatile compounds in rhizomes after transfer to the greenhouse

**DOI:** 10.1186/s12870-018-1358-6

**Published:** 2018-07-17

**Authors:** Rabia F. El-Hawaz, Mary H. Grace, Alan Janbey, Mary Ann Lila, Jeffrey W. Adelberg

**Affiliations:** 1 Department of Plant and Environmental Sciences, Clemson University, Clemson, SC 29634 USA; 2 Plants for Human Health Institute, North Carolina State University, Kannapolis, NC 28081 USA

## Correction

Following publication of the original article [1], the author reported a formatting error and an error in the figure caption. The original article has been corrected. The details of the errors are as follows:

1. In Table 2, The number in the second column and the forth row has to be (0.0267) instead of (0.0267 00101)! The number (0.0101) should be in the row 16th of the same column. The number in the last column and the third row has to be (0.0001) instead of 0.000.0001. The last row in the last column number had to be (0.666) instead of 0.660.666.

**Table 2:** The significant terms of the response surface models of GC-MS analysis in *Curcuma longa* L.35–1 rhizome

	Compounds (^a^)									
Significant model terms	(1)	(5)	(6)	(7)	(8)	(9)	(10)	(11)	(12)	Total
High-input Fertilizer	0.0001	0.0001	0.0001	0.0001	0.0001	0.0001	0.0001	0.0001		0.0001
Ca^2+^ mM	0.0267	0.0013	0.0059	0.0019	0.0478	0.0001	0.0042	0.0121	0.0395	0.0166
KNO_3_ mM						0.0079	0.0200			
Ca^2+^ × KNO_3_ mM		0.0312	0.0333	0.0104		0.0070	0.001	0.0076		0.0458
Mg^2+^ mM						0.0073				
Mg^2+^ × KNO_3_ mM						0.0003			0.0208	
Ca^2+^ × Mg^2+^ mM							0.013			
High-input × Buds/Vessel					0.0416					
High-input × PO_4_^3−^ mM						0.0083			0.0186	
High-input × Ca^2+^ mM					0.0422				0.0340	
High-input × KNO_3_ mM					0.0494					
Buds/Vessel× PO_4_^3−^ mM						0.0378				
(PO_4_^3−^ mM)^2^					0.0041	0.0060			0.0324	
(Ca^2+^ mM)^2^	0.0101									
*R*^*2*^ =	0.804	0.785	0.687	0.785	0.772	0.688	0.771	0.701	0.448	0.666

^a^(1) *β*-elemene, (5) *β*-elemenone, (6) isocurcumenol, (7) germacrone, (8) neocurdione, (9) curcumenone, (10) curcumenol isomer *I*, (11) curcumenol isomer *II*, and (12) methenolone
2. In the caption of Fig. 2 - “(a) The fed-batch was applied twice during the 5 month growth period. The volume of water and sucrose concentration was returned to the set point in the sucrose fed-batch subset (SF), additional to that the mineral concentrations were returned to the set point in the mineral-sucrose fed-batch subset NSF). (b) *Curcuma longa* L. 35-1 filled the 2.5 L bioreactor during 5 months with nutrient supplementation.” The word mineral has to be changed to nutrient.
